# *Cladophialophora bantiana* and *Nocardia farcinica* infection simultaneously occurring in a kidney transplant recipient: Case report and literature review

**DOI:** 10.1002/iid3.480

**Published:** 2021-06-15

**Authors:** Pedro Cortés, D. Jane Hata, Claudia Libertin, Diana M. Meza Villegas, Dana M. Harris

**Affiliations:** ^1^ Division of Community Internal Medicine Mayo Clinic Jacksonville Florida USA; ^2^ Division of Laboratory Medicine and Pathology Mayo Clinic Jacksonville Florida USA; ^3^ Division of Infectious Diseases Mayo Clinic Jacksonville Florida USA

**Keywords:** brain abscess, *Cladophialophora*, *Nocardia*, renal allograft

## Abstract

Solid organ transplant recipients are at increased risk of acquiring devastating infections with unusual pathogens. *Nocardia* are aerobic actinomycetes that affect the lungs, brain, skin and soft tissue. *Cladophialophora* species are dematiaceous fungi that overwhelmingly cause infections in the brain. Both organisms carry a high mortality rate. We present the first reported renal transplant case with *Cladophialophora bantiana* involving the renal allograft with concurrent invasive nocardiosis involving the lungs and brain.

## Graphical abstract

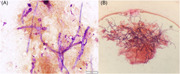

**Abbreviations**
CMV: cytomegalovirus
CNS: central nervous system
MCF: Mayo Clinic Florida
SOT: solid organ transplant
TMP/SMZ: trimethoprim/sulfamethoxazole

## 1 BACKGROUND

*Nocardia* is a ubiquitous gram‐positive, filamentous bacterium that is present in soil and water. This organism was first discovered in 1888 by Edmond Nocard after isolating it from cattle with farcy, hence *Nocardia farcinica*.^1^ Since then, microbiological studies have identified several dozens of species within this complex taxonomy.^2^ Despite its complexity, *Nocardia* only causes 500 to 1000 new infections in the United States each year.^3^

*Nocardia* primarily causes pulmonary, central nervous system (CNS), or soft tissue infections. In one study, 110 patients with invasive nocardiosis were identified over a 20‐year period.^4^ The rate of infection and mortality from nocardiosis was similar between transplant and non‐transplant patients. Still, most non‐transplant patients with nocardiosis were immunosuppressed, had chronic obstructive lung disease, chronic kidney disease or diabetes.^4^ The three main sites of infection were lung (73.6%), skin and soft tissue (23.6%), and brain (11.8%) with 20.9% of patients having disseminated nocardiosis.

*Cladophialophora* sp. are a genus of molds that inhabit living and dead material and are present pervasively in the environment.^5^ They are classified as dematiaceous fungi, meaning they produce a melanin‐like pigment in their cell walls and spores, giving them a black and brown complexion.^5^

*Cladophialophora* sp. have been associated with cerebral phaeohyphomycosis, a rare infection due to dematiaceous fungi affecting the CNS.^6^ It affects both immunocompromised and immunocompetent individuals.^5^ It has been reported worldwide, with most cases reported in Asian countries, especially India.^6^ Although brain abscesses account for 97.5% of cases with *Cladophialophora* sp., other possible sites of infection include pulmonary, skin and soft tissue.^7^, ^8^ Mortality from CNS involvement is high and is reported at 77.1% for immunocompromised patients.^6^ *Cladophialophora bantiana* is most often in association with CNS infections, brain infections, and abscesses due to its neurotrophic nature.^9^

### 1.1 Case presentation

A 34‐year‐old African American, cytomegalovirus (CMV)‐positive male with a history of end‐stage‐kidney‐disease (ESKD) from hypertensive nephrosclerosis underwent a CMV‐positive deceased donor renal transplantation with thymoglobulin induction in May 2015 at Mayo Clinic Florida (MCF), a major transplant center. His postoperative course was complicated by delayed graft function requiring postoperative dialysis for 10 days, followed by cellular rejection at 1 month after transplantation treated with high‐dose corticosteroids. CMV nephritis occurred a year later, which was successfully treated with intravenous (IV) valganciclovir. Then cellular rejection developed in August 2017, 2 years after transplantation, and was treated with high‐dose corticosteroids. His daily immunosuppressive regimen was tacrolimus, mycophenolate, and prednisone 10 mg for GFR 21 ml/min (creatinine 3.97 mg/dl). He was not compliant with clinic visits and eventually moved out of state.

In December 2018, he developed recurrent skin and soft tissue abscesses involving the left elbow and shoulder. He went to an outside hospital at another institution. The abscesses were incised and drained and grew *N. farcinica*. He was treated with linezolid for 2 weeks, but management was complicated with thrombocytopenia requiring a change to doxycycline planned for one year. In January 2019, the patient was found to have a fluid collection over the renal allograft site on the right lower quadrant. Drainage of the fluid grew *C. bantiana* and he was started on treatment with isavuconazonium. A drain was placed and removed one month later after an ultrasound showed resolution of the fluid collection. He was told that he was going to need a graft nephrectomy. On March 20, 2019, he returned to MCF for a second opinion regarding his management.

He complained of worsening abdominal pain over the allograft site and weight loss of 13 kg in 6 months on presentation. His renal function worsened with GFR < 15 ml/min (creatinine 6.24 mg/dl). A complete blood count revealed anemia with hemoglobin 6.9 g/dl (12.2–16.6 g/dl), MCV 81 (78–98 fl), leukopenia with a white blood cell count of 3 × 10^9^/L (3.4‐9.6×10^9^/L) and platelets 203 × 10^9^/L (135–217×10^9^/L). He took tacrolimus 14 mg twice daily, mycophenolate 1000 mg twice daily and prednisone 10 mg daily. A year prior at MCF, his hemoglobin was 9.1 g/dl, white blood cell count 3.3 × 10^9^/L, and platelets 175 × 10^9^/L. He took tacrolimus 4 mg twice daily, mycophenolate 1000 mg twice daily, and prednisone 10 mg daily. Upon hospital admission, the tacrolimus dose was decreased to half empirically until a tacrolimus level was drawn. The tacrolimus trough level was 7.2 ng/ml (5–15 ng/ml) the day after admission.

**Figure 1:**
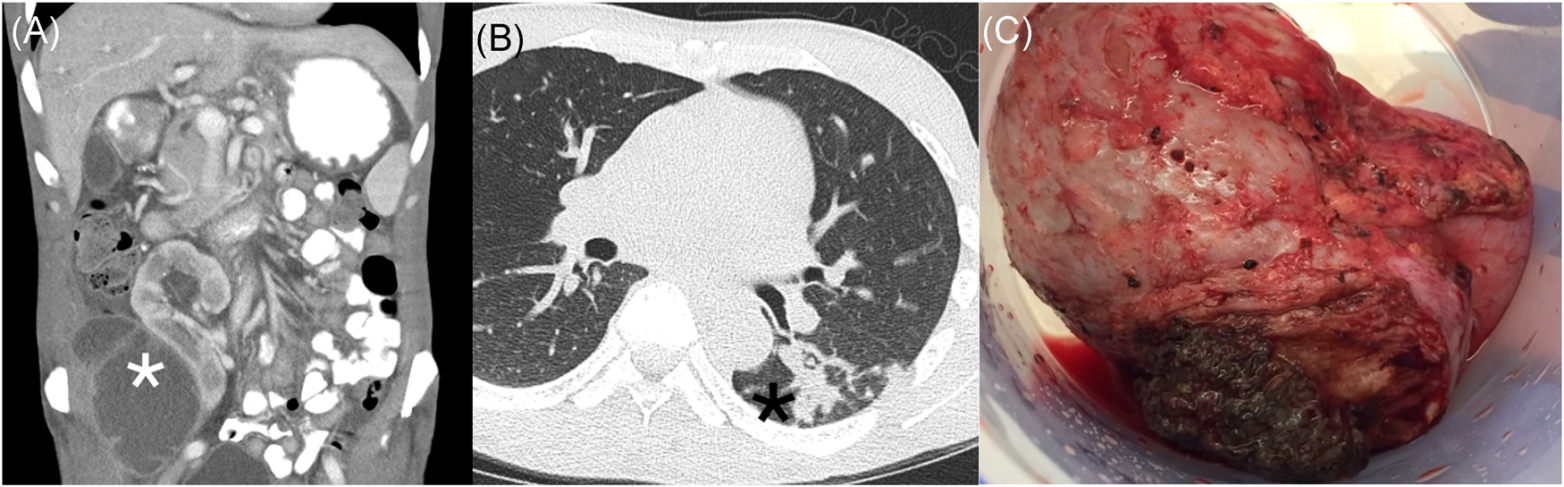
(A) Complex fluid collection lateral and inferolateral to transplanted pelvic kidney. (B) Nodular, ground‐glass opacities in the superior segment of left lower lobe. C. Right renal allograft following transplant nephrectomy. Cultures of biopsy tissue grew *Cladophialophora bantiana*

**Figure 2:**
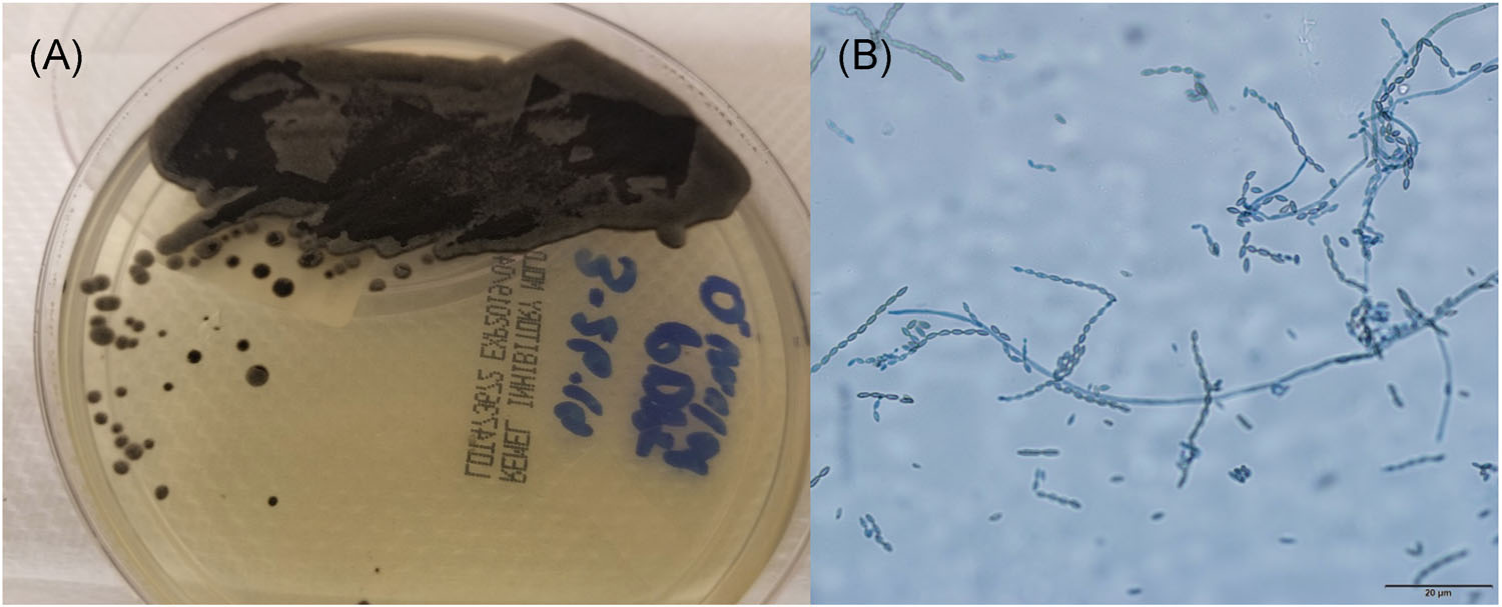
(A) Dematiaceous mold growing after 1 week of incubation at 30o C on inhibitory mold agar. (B) Lactophenol blue slide preparation (×10) of *Cladophialophora bantiana*. Note long conidia in chains with sparse branching

A renal ultrasound revealed a complex peri‐transplant fluid collection measuring 12 cm. A computer tomography (CT) abdomen and pelvis with IV contrast confirmed the fluid collection compatible with liquefied hematoma or a possible abscess. A percutaneous drain was placed (Figure 1A). Cultures from the peri‐nephric fluid grew a dematiaceous mold after 7 days which was identified by morphology and ITS sequencing as *C. bantiana* (Figure 2). Based on fungal susceptibility testing results, Isavuconazonium was changed to liposomal amphotericin B and voriconazole was begun. Doxycycline was continued.

Within the first week of hospitalization, the patient developed acute epistaxis and disclosed that he had had a dry cough for several weeks. A CT chest was performed, which showed nodular ground‐glass opacities with associated air bronchograms within the left lower lobe superior segment compatible with an infectious process (Figure 1B).

**Figure 3:**
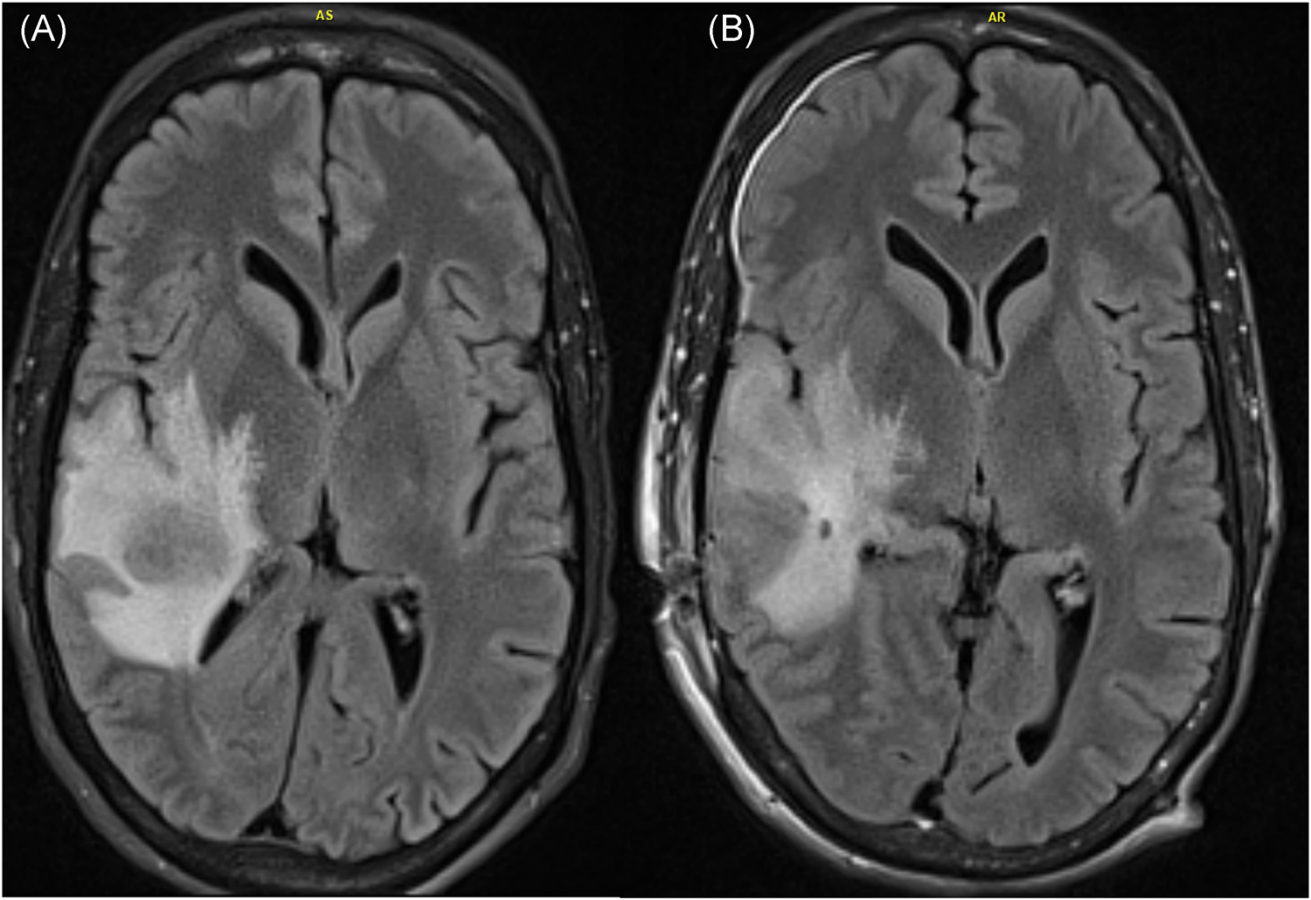
(A) T2 FLAIR of lobulated mass in posterior superior aspect of right superior temporal gyrus with peripheral decreased T2 signal intensity with associated mass effect, most consisted with abscess. (B) Complete drainage of abscess and resection of abscess capsule after craniotomy

A CT of the brain and sinuses was performed, which showed a 2.4 × 1.8 cm right parietotemporal mass with a hyperattenuating rim and central low‐density matter. There was significant vasogenic edema in the right parietal lobe extending into the right temporal lobe with a 4 mm leftward midline shift at the septum pellucidum. Magnetic resonance imaging (MRI) of the brain confirmed the findings, showing decreased T2 signal intensity and significant central restricted diffusion of mass, concerning for an abscess (Figure 3A). Given the pulmonary and CNS findings, IV trimethoprim/sulfamethoxazole (TMP/SMZ) and meropenem were initiated.

**Figure 4:**
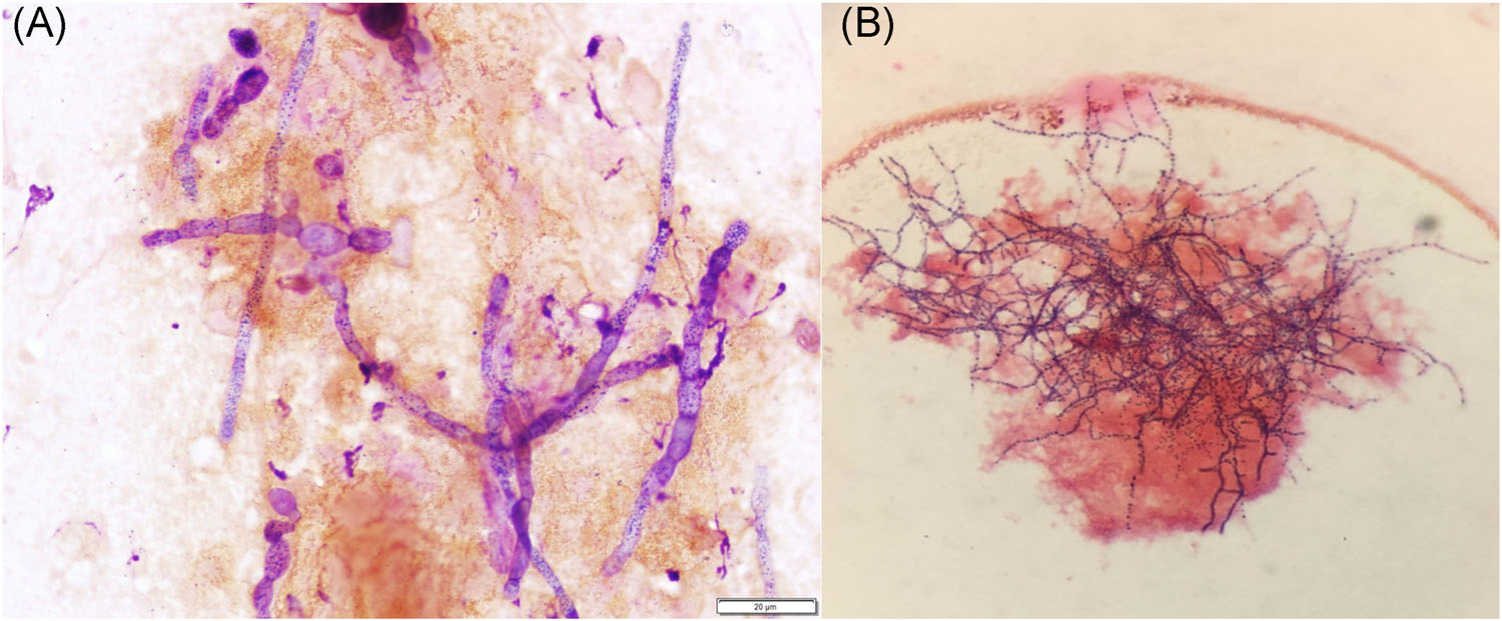
(A) Touch prep of kidney tissue biopsy showing *Cladophialophora bantiana*. Gram stain, ×10. (B) Gram stain (×10) showing beaded and branching gram‐positive bacilli consistent with *Nocardia farcinica*

Neurosurgery performed a right temporal craniotomy. Postoperatively, an MRI showed complete drainage of the abscess (Figure 3B). Cultures from the brain grew small yellow‐orange dry colonies identified as *N. farcinica* by MALDI‐TOF mass spectrometry. Susceptibility testing indicated resistance to cefepime, ceftriaxone, clarithromycin and tobramycin (Figure 4B). Two days after craniotomy, he underwent a right transplant nephrectomy with abscess drainage and debridement of muscle tissue (Figure 1C). Numerous dematiaceous hyphae were noted on fungal smear of the renal specimen. Culture of tissue from the renal allograft grew grew *C. bantiana* (Figure 4A).

Other notable infectious studies included negative plasma BK virus PCR, CMV DNA PCR, HIV‐1 p24 antigen and antibody screen, and HIV‐2 antibody screen. During his hospitalization, his transplant immunosuppressant regimen was tacrolimus 7 mg orally twice daily, mycophenolate 1,000 mg orally twice daily, and prednisone 10 mg daily.

Despite IV fluids and treatment of infections, the patient's renal function worsened, requiring the initiation of hemodialysis (HD). The patient was discharged after 2 weeks on April 4, 2019 to continue liposomal amphotericin B and voriconazole with trough drug monitoring every 2 weeks. Also, meropenem was prescribed to complete 8 weeks of therapy, and doxycycline and TMP/SMZ were to be continued for up to a year. Repeat imaging of the brain, chest, and abdomen was planned for 6 weeks. Unfortunately, the patient did not attend his subsequent outpatient appointments and was lost to follow up.

## 2 DISCUSSION

The case represents the first report of *C. bantiana* causing a soft tissue infection at the renal allograft site. Concurrently, the patient had CNS involvement from *Nocardia*. The pulmonary findings appeared to be infectious and may have been secondary to either *Nocardia* or *Cladophialophora*. The pathogen was not identified given the high risk of endobronchial biopsy.

The combination of both organisms causing separate but unique infections is rare. Our case is the second one where the two pathogens, *Nocardia* and *Cladophialophora*, caused simultaneous infections in a transplant recipient.^10^ In the case by Khaliq et al.^10^ the patient presented with ataxia and confusion, concerning for CNS involvement. He was found to have a brain abscess caused by *Cladophialophora* and multifocal pneumonia caused by *Nocardia*. The present case differs as the brain abscess was secondary to *Nocardia*, and *Cladophialophora* caused infection at a non‐CNS site.

In the literature, there have been multiple reports of cases of brain abscesses caused by *Cladophialophora* sp. in solid organ transplant (SOT) recipients.^11^, ^12^, ^13^, ^14^, ^15^, ^16^, ^17^, ^18^, ^19^, ^20^ Non‐CNS involvement by *Cladophialophora* sp. is very sporadic regardless of immunocompromised status, and includes skin and pulmonary involvement.^8^, ^21^ CNS involvement by *Cladophialophora* sp. is more prevalent in immunocompetent individuals and has a propensity toward males.^7^ Interestingly, the neurotropism of *Cladophialophora* sp. has been attributed to its melanin‐like pigment similar to melanoma's predisposition to metastasize to the brain.^22^ It is important to note that due to the neurotrophic nature of *C. bantiana*, attempts to culture the fungus must be performed with enhanced biosafety measures to prevent possible exposure to laboratory personnel.

In the literature, there have been three other cases of non‐CNS involvement by *Cladophialophora* sp. in SOT recipients (Table 1). An isolated non‐CNS infection caused by *Cladophialophora* sp. only occurred in case one.^23^ CT imaging of the brain and sinuses excluded concurrent CNS infection. The two other cases initially presented with a non‐CNS site of infection and were found to have CNS abscesses after several days of being hospitalized.^24^, ^25^ The authors hypothesized the infection disseminated via hematogenous spread to the brain based on the multiple brain abscesses seen on imaging.

**Table 1:** Cases of non‐CNS *Cladophialophora* infections in transplant recipients

Case	Transplant	Age/Sex	Induction regimen	Immunosuppression regimen	Chief complaint	Site of infection	Diagnosis	Treatment	Outcome	Year published
1 [23]	Lung	59 M	Thymoglobulin at 200 mg/d for 15 days	Cyclosporine (trough 80–120 ng/ml), prednisone 0.25 kg/mg/d	DOE	Pulmonary	Respiratory culture grew *C. boppii*	Liposomal, nebulized, and endobronchial amphotericin B	Progression to cavitations, died 8 months after transplantation	2009
2 [24]	Renal	34 F	Unknown	Tacrolimus, mycophenolate mofetil, low‐dose methylprednisolone	Unknown	Initially left proximal tibia; then possible CNS	Deep bone biopsy grew *C. bantiana*	Debridement, amputation, voriconazole, liposomal amphotericin B	Improvement after lengthy disease course complicated by renal failure	2016
3 [25]	Heart	57 M	Thymoglobulin	Prednisolone, azathioprine, tacrolimus	Shoulder wound	Initially cutaneous; then CNS	Wound and brain culture grew *C. bantiana*	Initially liposomal amphotericin B, then itraconazole	Deteriorated one month after diagnosis and died	2002

The present case is unique and educational for several reasons. First, the patient presented almost concurrently with *Nocardia* and *Cladophialophora* infections. This likely resulted from intensive immunosuppression from advancing renal failure, high dose tacrolimus, mycophenolate and corticosteroids. It is known that the immunosuppressant medications the patient was taking (corticosteroids, tacrolimus, mycophenolate) suppress T‐cell function and predispose to *Nocardia* sp. infections which occurs late after an organ transplant (12–34 months).^4^, ^26^, ^27^, ^28^ *N. farcinica*, is one of the most commonly isolated species along with *Nocardia nova* complex and *Nocardia cyriacigeorgica*.^29^ Cutaneous *N. farcinica* may have disseminated to the lung and brain explaining his cough symptoms and lung imaging findings. *N. farcinica* has a predilection on the brain.^26^ The patient did not have any CNS symptoms upon presentation, consistent with other studies of transplant patients in which only half of patients with CNS *Nocardia* had neurological symptoms.^26^ Most *Nocardia* sp. are susceptible to TMP/SMZ, linezolid and amikacin, but the standard of practice recommends combination antibiotics for empiric therapy due to the seriousness of the infection. Of note, only 2% of species of *N. farcinica* are susceptible to doxycycline.^4^, ^29^ One year mortality in solid organ transplant patients is 15%–20%.^30^ The worsening anemia and leukopenia of the patient were the result of the high dose immunosuppression on the bone marrow.

Second, the peri‐nephric fluid collection with *C. bantiana* should have been appropriately treated with drain placement and isavuconazole at the outside hospital. Isovuconazole has good susceptibility for *Cladophialophora*, and fungal abscess treatment includes drainage and surgical debridement.^9^, ^22^ Both treatment modalities were given to the patient. In vivo studies have shown sensitivity to amphotericin B, itraconazole, voriconazole and micafungin.^21^ Reasons that the management failed may include the possibility that the drain was removed early, the fungus already invaded the kidney, the patient was not compliant with taking the isavuconazole or follow up to identify relapsed infection did not occur. Most interestingly, *Cladophialophora* caused a non‐CNS infection at the site of renal allograft. The entry may have been the lungs with hematogenous spread to the renal allograft, and given time, it is reasonable to postulate the hematogenous spread of *C. bantiana* to involve the brain, along with the *N. farcinica* isolated eventually. Nonetheless, the isolation of a localized *Nocardia* or *Cladophialophora* infection always warrants evaluation of the CNS as it is the most common site of extrapulmonary involvement.^31^, ^32^ Despite treatment, mortality from brain abscess caused by *Cladophialophora* sp., remains exceptionally high, upwards to 77.1% in immunocompromised patients.^6^ The present patient was treated with a transplant nephrectomy and liposomal amphotericin B and voriconazole to complete an 8‐week course. His outcome is not known.

## 3 CONCLUSION

SOT recipients are at increased risk of developing disseminated infections with unusual organisms with high mortality. We must maintain a high index of suspicion especially for fungal and less common bacterial infections to properly diagnose, treat, and follow these patients. As described in this case, due to severe immunosuppression, patients may present with more than one kind of infection. Herein, we present the first case in the literature of a patient with *C. bantiana* involving the transplanted organ, while having simultaneous invasive Nocardiosis involving the brain and likely the lungs. *Nocardia* sp. infections present 1 to 3 years after transplant and the most commonly affected organ is the lung. Hematogenous dissemination may occur from the lung or skin to the brain. Empiric treatment requires a combination of antibiotics. *Cladophialophora* sp. almost exclusively causes CNS involvements in the form of multifocal brain abscesses. Non‐CNS involvement by *Cladophialophora* sp. is extremely rare. The mainstay of treatment is broad‐spectrum antifungals and surgical debridement of infected tissue.

## AUTHOR CONTRIBUTIONS

Pedro Cortés collected the clinical data, composed the figures and table and drafted the manuscript; Claudia Libertin reviewed the article for accuracy and provided expert infectious diseases input; D. Jane Hata provided the pathological information and expert microbiology input; Diana M. Meza Villegas provided the pathological information and expert microbiology input; Dana M. Harris reviewed the article for accuracy and provided expert infectious diseases input.

## CONFLICT OF INTERESTS

The authors declare that there are no conflict of interests.
